# CRISPR-Cas Immunity against Phages: Its Effects on the Evolution and Survival of Bacterial Pathogens

**DOI:** 10.1371/journal.ppat.1003765

**Published:** 2013-12-12

**Authors:** Luciano A. Marraffini

**Affiliations:** Laboratory of Bacteriology, The Rockefeller University, New York, New York, United States of America; Duke University Medical Center, United States of America

## Introduction

**Figure 1:**
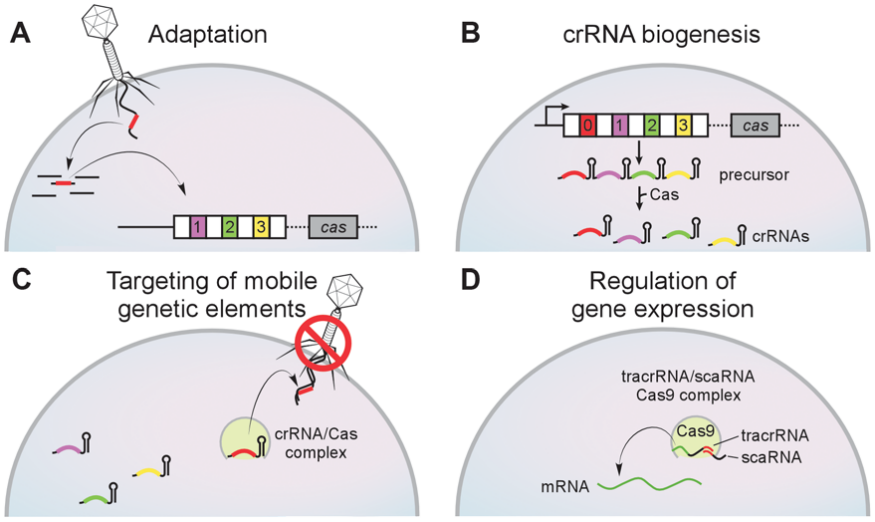
The CRISPR immunity pathway. CRISPR loci contain clusters of repeats (white boxes) and spacers (colored boxes) that are flanked CRISPR-associated (*cas*) genes. (**A**) During adaptation new spacers derived from the genome of the invading virus are incorporated into the CRISPR array by an unknown mechanism. Repeat duplication is also required. (**B**) During crRNA biogenesis a CRISPR precursor transcript is processed by Cas endoribonucleases within repeat sequences to generate small crRNAs. (**C**) During targeting the match between the crRNA spacer and target sequences specifies the nucleolytic cleavage of invading mobile genetic elements such as viruses and plasmids. (**D**) In the CRISPR-Cas system of *F. novicida*, the tracrRNA (a small RNA mediated in crRNA biogenesis in this system) contains homology to the BLP (bacterial lipoprotein) transcript. The base-pair interaction between the tracrRNA and the BLP mRNA (mediated also by another small RNA, the scaRNA, and the nuclease Cas9) regulates the expression of this immunomodulatory lipoprotein.

Clustered regularly interspaced short palindromic repeats (CRISPR) loci are arrays of short repeats separated by equally short “spacer” sequences [1]–[3]. Along with the CRISPR-associated (*cas*) genes, they encode an adaptive immune system of archaea and bacteria that protects the cell against viral infection [4]. Remarkably, this system is capable of inserting a short piece of an infecting viral genome as a spacer in the CRISPR array [4], [5] (Figure 1A). The spacer sequence is transcribed and processed to generate a small antisense RNA (the CRISPR RNA or crRNA) (Figure 1B) [6] that is used as a guide for the recognition and destruction of the invader in subsequent infections (Figure 1C) [7]. Thus, spacer acquisition immunizes the bacterium and its progeny against the virus from which it was taken. Because spacers are incorporated in sequential order, CRISPR loci reflect the history of viral infection of the host. Cas proteins participate in all the different steps of this pathway, namely the insertion of spacer sequences into the CRISPR array [8], [9], the biogenesis of crRNAs [10], [11], and the destruction of the infecting viral genome [12], [13].

## Distribution of CRISPR-Cas Loci among Bacterial Pathogens

In spite of the unique role that CRISPR-Cas loci play in antiviral defense, they are not universal. To date, the CRISPR database [14], a webtool that determines the presence of CRISPR arrays in completed genomes, indicates that 119/141 archaeal (84%) and 1012/2113 bacterial (48%) genomes contain CRISPR loci. In bacteria, there are species in which all strains have CRISPR loci, some in which only some strains have these loci, and species without strains having CRISPR loci. Therefore it is not possible to determine unequivocally that lack of CRISPR in certain strains or species is due to loss of these loci. However, because CRISPR sequences are spread thorough horizontal gene transfer [15], [16] and can be easily lost [17]–[20], it has been hypothesized recently that CRISPR are in a constant state of flux and can appear and disappear depending on the selective forces of the environment [20]. The same type of uneven distribution is found when we look at the presence of CRISPR loci in bacterial pathogens in the CRISPR database (http://crispr.u-psud.fr/crispr/).

## CRISPR-Cas Systems as a Barrier to Horizontal Gene Transfer

While most of the spacers with matches on GenBank target prokaryotic viruses (phages), there is a still an important fraction that match other targets. A recent study looked at all the spacer hits of archaeal CRISPR loci [21] and reported that 40% of them matched phage sequences. The remaining 60% matched other mobile genetic elements such as conjugative plasmids and transposons (22%), CRISPR-Cas loci (18%), and other genes not associated with mobile elements (hypothetical ORFs and housekeeping genes, 20%). Although an equally extensive study has not been performed with bacterial CRISPR spacers, partial analysis suggests a similar distribution [22], [23]. While the presence of antiphage spacers is key for the defense of the cell, the origin and function of these nonphage targeting spacers is obscure. How are these spacers acquired? One possibility is that these sequences are inserted into CRISPR loci during the transfer of foreign genetic material that commonly occurs between prokaryotes, also known as horizontal gene transfer (HGT) [24]. In this scenario, non-antiphage spacers are acquired during bacteriophage transduction, plasmid conjugation, or upon the uptake of foreign DNA during natural transformation. Alternatively, spacer acquisition only occurs as an adaptive response to phage infection and the nonphage targeting spacers are acquired only from phage transducing particles [25]. Regardless of whether the diversity of the CRISPR spacer repertoire is generated by accident or not, the fact that CRISPR loci can target all sorts of genetic material argues that these loci constitute a barrier against the horizontal transfer of genes and accessory genetic elements. Indeed, CRISPR interference has been shown experimentally to prevent the acquisition of conjugative plasmids [26], integrative conjugative elements [27], and environmental DNA by natural transformation [17], [28]. What is even more puzzling is the function, if any, of these nonphage targeting spacers. Plasmid targeting could eliminate the burden of additional replicating elements inside the cell, and the targeting of housekeeping genes could provide a regulatory function for these spacers. However, plasmids, mobile genetic elements, and foreign genes can provide a fitness advantage or even be essential for survival (e.g., antibiotic resistance genes).

## Implications of CRISPR-Mediated Targeting of Mobile Genetic Elements in Bacterial Pathogens

HGT is the major source of genetic diversity for bacterial evolution [24]. In the past century, the introduction of modern antibacterial therapies has accelerated the evolution of pathogens. While it is clear that HGT has played a central role in the spread of virulence factors and antimicrobial resistance genes [29], [30], only a few studies have addressed whether and how CRISPR loci, owing to their potential to regulate HGT, impact the evolution of pathogens. One of these studies investigated the relationship between the CRISPR loci and the prophage content of group A streptococci (GAS, *Streptococcus pyogenes*), one of the most prevalent human bacterial pathogens. These organisms contain between two to eight prophages, each encoding at least one virulence factor [31]. Bioinformatic analysis revealed that seven of the 13 available GAS genomes contain CRISPR-Cas loci and that there is a mutually exclusive relationship between CRISPR spacer sequences and their prophage targets [32]. This suggests that there is a dynamic relationship between *S. pyogenes*, its phages, and its CRISPR loci that results in the selection of strains with increased pathogenic adaptations. CRISPR-Cas loci also can impact the spread of antibiotic resistance. Pathogenic staphylococci have acquired resistance to all known antibiotics [33], primarily through the acquisition of conjugative plasmids carrying resistance genes [30]. *Staphylococcus epidermidis* RP62a is a clinical isolate containing a CRISPR-Cas system with a spacer matching all staphylococcal conjugative plasmids sequenced to date [34]. This spacer provides immunity against the conjugative transfer of these plasmids [26], thereby preventing the acquisition of the antibiotic resistances that they carry. Therefore CRISPR loci could control the dissemination of antibiotic resistance in staphylococci. This does not seem to be the case for *Escherichia coli*. A study of a collection of 263 natural *E. coli* isolates from human and animal hosts revealed that CRISPR loci neither match plasmid sequences nor correlate with the presence or absence of plasmids or antibiotic resistance genes [35].

## Loss of CRISPR-Cas Loci in Bacterial Pathogens

CRISPR immunity against conjugative plasmids would compromise the survival of *S. epidermidis* RP62a, and other staphylococci carrying similar CRISPR-Cas systems [36], [37] in hospital or other settings where antibiotics are used. A recent study [20] looked for the transfer of the mupirocin-resistant conjugative plasmid pG0400 into *S. epidermidis* to determine if a CRISPR-Cas system and its target could coexist to prevent this potentially detrimental antiplasmid activity of CRISPR immunity. Immunity against the plasmid was found to decrease the transfer efficiency by about four orders of magnitude but not absolute. Transconjugants that evaded CRISPR attack were analyzed only to find that in all cases they harbored preexisting CRISPR-Cas mutations that allowed plasmid transfer. Loss of CRISPR-Cas loci upon transfer of antibiotic resistant plasmids also seems to occur in enterococci. A screen of 45 strains of *Enterococcus faecalis* showed a correlation between the presence of CRISPR-Cas loci and antibiotic resistance genes [38]. Finally, another recent study explored the consequences of CRISPR targeting of *Streptococcus pneumoniae* capsule genes, essential for pneumococcal infection. During infection, natural transformation of capsule genes allows nonencapsulated, avirulent pneumococci to become encapsulated and kill the mice [39]. A CRISPR-Cas targeting a specific capsule gene was engineered into nonencapsulated *S. pneumoniae* and used to infect mice in the presence of heat-killed encapsulated pneumococci [17]. Horizontal transfer of capsule genes from heat-killed cells into live, nonencapsulated bacteria was prevented by CRISPR immunity, resulting in the survival of mice. The occasional mice that succumbed to pneumococcal infection, however, contained encapsulated bacteria carrying inactivating mutations in the engineered CRISPR locus. These and other results [18], [19] suggest that CRISPR loci and their targets cannot coexist in the same cell. In the case of strong environmental selection of a targeted gene or mobile element, only CRISPR mutants survive. This is a possible explanation for the lack of CRISPR in *S. pneumoniae* and *S. aureus*, two notoriously fast-evolving pathogens, but also in other bacteria and archaea that lack this immune system.

## A Direct Role for CRISPR-Cas Systems in Bacterial Pathogenesis

While the reasons for the absence of CRISPR-Cas loci in some fast-evolving pathogens remain a matter of speculation, recent evidence showed that these loci can also promote pathogenesis. A study in *Legionella pneumophila* showed that *cas2*, a gene involved in the acquisition of new spacers, is required for the propagation of this pathogen inside amoebae hosts [40], although it is not clear what the function of this gene is during growth. More compelling evidence is found in the intracellular pathogen *Francisella novicida*. In this bacterium, *cas9* is a CRISPR-associated dsDNA nuclease that requires, in addition to the crRNA guide, a tracrRNA (*trans*-activating crRNA) for cleavage of the invader genome [41], [42]. It was found recently that *cas9* is required to repress the production of a bacterial lipoprotein (BLP), a toll-like receptor 2 (TLR2) ligand that induces an innate immune inflammatory response [43]. Repression is independent of the crRNA guides, but requires the tracrRNA and a new small CRISPR-associated RNA (scaRNA) with complementarity to the tracrRNA [44], [45]. The tracrRNA, in turn, contains an ∼85 nt region with partial complementarity to the 3′-end of the BLP messenger, an interaction that leads to the BLP mRNA degradation through an unknown mechanism. This CRISPR-mediated regulation of BLP expression allows *F. novicida* to evade the host's immune response. A similar mechanism seems to be in place in other pathogens as well: deletion of *cas9* in *Neisseria meningitidis* affected virulence traits such as adherence to and invasion of human epithelial cells [44], and inactivation of *cas9* in *Campylobacter jejuni* resulted in reduced virulence [46]. While the predominance of tracrRNA/scaRNA-mediated regulation remains to be investigated, its existence suggests that CRISPR-Cas loci can be easily converted into regulatory elements that enhance bacterial pathogenesis.

## Conclusions

Clearly CRISPR-Cas systems can both prevent the evolution of pathogenesis, and thus be lost or mutated in bacterial pathogens, but also be co-opted by the pathogen to increase virulence. This will depend of a series of factors: whether other antiphage systems can fulfill the function of the lost CRISPR-Cas system, whether the pathogen relies heavily on HGT for survival, and whether the CRISPR-Cas system can be easily converted into a regulator of gene expression. In the face of the lateral transfer of CRISPR systems, the repression of gene expression by CRISPR provides another level of selection for the maintenance of these systems. While the repression of BLP provides a selectable advantage for *Francisella*, the accidental repression of essential genes (which could be produced by a fortuitous base-pairing of the tracrRNA and an essential transcript) will select against the lateral transfer of some CRISPR-Cas systems into certain hosts. In the future, the application of DNA sequencing technologies to epidemiological studies will allow us to measure correlations between the flux of CRISPR-Cas loci and the acquisition of antibiotic-resistance plasmids and pathogenicity islands or genes, thus allowing us to measure the effect of CRISPR on the emergence of virulence. On the other hand, the importance of CRISPR for pathogenesis provides a new target for antimicrobials with anti-CRISPR activity. Interestingly, phages already found such anti-CRISPR compounds for us: as part of their arms race with bacteria, phages have developed CRISPR inhibitors [47]. The intersection between CRISPR biology and bacterial pathogenesis is a new and exciting research area that is only beginning to be explored.
